# Preoperative cancer antigen-125 levels as a predictor of recurrence in early-stage endometrial cancer

**DOI:** 10.1590/1806-9282.20231115

**Published:** 2024-05-20

**Authors:** Anil Erturk, Elmas Korkmaz, Zeynep Arslantas, Sena Bekdemir, Nergis Kender Erturk

**Affiliations:** 1 University of Health Sciences, Bursa Yuksek Ihtisas Educational and Research Hospital, Department of Obstetrics and Gynecology – Bursa, Turkey.; 2 Kartal Dr. Lutfi Kirdar Educational and Research Hospital, Department of Obstetrics and Gynecology – İstanbul, Turkey.

**Keywords:** Endometrial cancer, Endometrioid adenocarcinoma, Prognosis, CA-125 antigen, Recurrence

## Abstract

**OBJECTIVE:**

Endometrial cancer is the most common gynecological cancer in developed countries, with a majority of cases being low-grade endometrioid endometrial cancer. Identifying risk factors for disease recurrence and poor prognosis is critical. This study aimed to assess the correlation between preoperative cancer antigen-125 levels and disease recurrence in early-stage endometrioid endometrial cancer patients.

**METHODS:**

The study was a retrospective analysis of 217 patients diagnosed with endometrioid endometrial cancer who underwent surgical treatment at a university-affiliated tertiary hospital between 2016 and 2022. Patients were divided into two groups based on their preoperative cancer antigen-125 levels and compared with clinicopathological findings and disease recurrence. Disease-free survival rates were calculated, and logistic regression analysis was performed to determine independent factors affecting disease-free survival.

**RESULTS:**

The mean age of patients was 61.59±0.75 years, and the mean follow-up time was 36.95±1.18 months. The mean cancer antigen-125 level was 27.80±37.81 IU/mL. The recurrence rate was significantly higher in the group with elevated cancer antigen-125 levels (p=0.025). Disease-free survival was lower in patients with elevated cancer antigen-125 compared with those with normal levels (p=0.005). Logistic regression analysis revealed that elevated cancer antigen-125 levels were associated with disease recurrence (OR: 3.43, 95%CI 1.13–10.37, p=0.029).

**CONCLUSION:**

The findings of this study suggest that preoperative cancer antigen-125 levels can be used as a predictor of disease recurrence in early-stage endometrioid endometrial cancer patients. cancer antigen-125 levels may be a useful tool for risk stratification and patient management in endometrial cancer.

## INTRODUCTION

Endometrial cancer (EC) is the most common gynecological cancer in developed countries^1^. The most common type of EC is endometrioid EC (EEC), which accounts for 75–80% of cases. Most EECs are low grade (grade 1–2), diagnosed at an early stage, and have a good prognosis^2^, but up to 7% of patients may still be at risk of disease-related mortality^3^. Considering that the majority of patients are in the low-grade EEC group, the number of disease-related deaths can be considered quite high. Additionally, recurrence in early-stage EC can be as high as 15–20%^4^. Therefore, it is crucial to identify patients at risk of poor prognosis before surgery.

Patients’ age, tumor size, tumor grade, histological type, and lymphovascular space involvement (LVSI) have been identified as risk factors for poor prognosis in ECs^5,6^. Preoperative cancer antigen-125 (CA-125) levels have also been linked to disease recurrence^7^. While an elevation in serum CA-125 levels has been found to be correlated with advanced-stage EECs, its role in early-stage EECs is still a topic of debate^8-10^.

In this study, our goal was to assess the correlation between preoperative CA-125 levels and disease recurrence in early-stage EEC patients.

## METHODS

Patients diagnosed with EC who underwent surgical treatment at a university-affiliated tertiary hospital between January 2016 and March 2022 were analyzed retrospectively after obtaining approval from the local ethics committee (2011-KAEK-25 2022-11/06). This study was conducted in accordance with the Declaration of Helsinki. The sociodemographic characteristics, preoperative CA-125 levels, surgery reports, histopathology results, and postoperative follow-up data of the patients were reviewed from electronic/archival files. A total of 217 patients were examined. Patients with non-endometrioid type adenocarcinomas (n=11), high-stage endometrioid cancers (n=13), no preoperative CA-125 levels (n=32), other concurrent cancers, pelvic endometriosis or adenomyosis or adnexal mass (n=9), follow-up examinations at another center (n=18), previous chemo-radiotherapy (n=9), or incomplete data (n=27) were excluded from the study. The final study population (n=167) included the International Federation of Gynecology and Obstetrics (FIGO) stage 1–2 EEC diagnosed and operated for the first time at our hospital.

Patients were divided into two groups based on their preoperative CA-125 levels: those with normal levels (<35 IU/mL) and those with elevated levels (≥35 IU/mL), and were compared against clinicopathological findings and disease recurrence.

All the patients underwent surgical staging according to FIGO classification^11^, which included total hysterectomy and bilateral salpingo-oophorectomy. Selective systemic pelvic-paraaortic lymphadenectomy was performed based on intraoperative frozen section findings using Mayo-Clinic criteria^12^. All the specimens were evaluated by gynecologic pathologists in our institution. The final histopathology reports included information on histological grade and type, myometrial invasion (MI), cervical invasion, LVSI, and lymph node metastasis status. The administration of adjuvant therapy was determined by a team of experts from multiple disciplines^13^. Recurrence was diagnosed by clinicians using physical examination and imaging reports. Disease-free survival (DFS) was defined as the time from surgery to the first recurrence of the disease.

Statistical analysis was conducted using SPSS version 23 (SPSS Inc., Chicago, IL, USA). The Shapiro-Wilk test was used to determine the normality of the variables. Non-parametric continuous data were compared using the Mann-Whitney U test, and categorical data were analyzed using the chi-square test. The Kaplan-Meier survival analysis was used to calculate DFS in patients based on their preoperative CA-125 levels. Logistic regression analysis was performed to identify independent factors associated with disease recurrence. A p<0.05 was considered statistically significant.

## RESULTS

The mean age of patients was 61.59±0.75 years, with a range of 36–86 years. The mean BMI was 35.97±0.31 kg/m^2^. The mean follow-up time was 36.95±1.18 months, with a range of 12–66 months. Complete surgical staging, including pelvic-paraaortic lymphadenectomy, was performed on 88.0% (n=147) of patients, while the remaining 12.0% (n=20) did not undergo lymphadenectomy. The mean number of lymph nodes removed was 49.37±0.94, with a range of 27–95 nodes. The mean preoperative CA-125 level was 27.80±37.81 IU/mL, with a range of 0.5–291.

In this study, 167 patients were evaluated, of which 125 (74.9%) had normal preoperative CA-125 values and 42 (25.1%) had elevated CA-125 levels. The demographic and clinical characteristics of the groups are presented in Table 1. The groups were comparable in terms of age, BMI, follow-up periods, and menopausal status (Table 1). No significant differences were observed between the groups with regard to tumor grade, MI, LVSI, and tumor stage (Table 1). Disease recurrence was significantly higher in the elevated CA-125 group compared with the normal CA-125 group (21.4 vs. 8.0%, p=0.025) (Table 1).

**Table 1:** Baseline characteristics of patients according to preoperative cancer antigen-125 level.

		CA-125 normal	CA-125 elevated	p
		(n=125)	(n=42)	
Age (years)*		61.23±10.09	62.69±8.97	0.502
BMI (kg/m^2^)*		36.15±4.28	35.45±3.39	0.667
Gravida		3 (0–8)	3 (0–9)	0.129
Follow-up (m)		39 (12–66)	32 (12–66)	0.156
CA-125 (IU/mL)*		13.04±5.90	71.74±55.15	<0.001
Menopause status				
	Premenopause	107 (85.6)	37 (88.1)	0.800
	Postmenopause	18 (14.4)	5 (11.9)	
Histological grade				
	G1	66 (52.8)	18 (42.9)	0.208
	G2	43 (34.4)	16 (38.1)	
	G3	16 (12.8)	8 (19.0)	
Tumor size				
	≤2 cm	18 (14.4)	7 (16.7)	0.803
	>2 cm	107 (85.6)	35 (83.3)	
MI				
	<50%	83 (66.4)	23 (54.8)	0.197
	≥50%	42 (33.6)	19 (45.2)	
	Cervical stromal invasion positivity	4 (3.2)	2 (4.8)	0.642
	LVSI positive	11 (8.8)	3 (7.1)	0.738
FIGO stage				
	I	121 (96.8)	40 (95.2)	0.642
	II	4 (3.2)	2 (4.8)	
Recurrence				
	Yes	10 (8.0)	9 (21.4)	0.025
	No	115 (92.0)	33 (78.6)	

Values are given median (min–max) or number (%), unless otherwise specified. Mann-Whitney U test or chi-square test was performed. p<0.05 was significant.

* Values are given as mean±SD.

Y: years; BMI: body mass index; m: months; G: grade; MI: myometrial invasion; LVSI: lymphovascular space involvement; FIGO: International Federation of Gynecology and Obstetrics.

**Figure 1:**
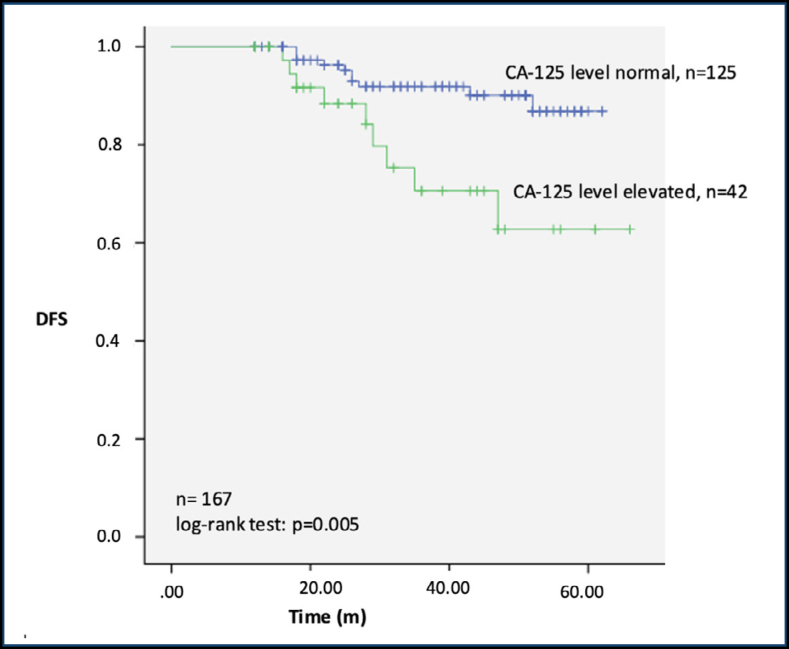
Kaplan-Meier survival analyses for disease-free survival in patients according to preoperative serum cancer antigen-125 levels. DFS: disease-free survival; m: months.

According to Kaplan-Meier analysis, DFS was significantly lower in the elevated CA-125 group compared with the normal CA-125 group (p=0.005) (Figure 1).

A logistic regression analysis was performed to identify factors associated with disease recurrence. The model included patient age, BMI, tumor grade, tumor size, LVSI, and elevated CA-125 levels. The results showed that this model was found to be significantly associated with disease recurrence (p<0.001, R^2^=0.22). LVSI positivity and elevated CA-125 levels were found to be significant independent prognosticators for disease recurrence (OR:8.64, 95%CI 2.18–34.19, p=0.002 and OR:3.43, 95%CI 1.13–10.37, p=0.029, respectively).

## DISCUSSION

In this study, we found that patients with preoperative CA-125 level ≥35 IU/mL had significantly higher rates of disease recurrence in EECs. The DFS in stage 1–2 EECs was found to be linked to preoperative CA-125 levels. Additionally, we determined that both an elevated preoperative CA-125 level and LVSI positivity were significant independent risk factors for disease recurrence in early-stage EECs.

The CA-125 antigen is a large transmembrane glycoprotein found in the cells of the pericardium, pleura, peritoneum, fallopian tube, and endometrial and endocervical tissues^14^. It is mostly used to monitor epithelial ovarian cancer^15^. Although there is evidence suggesting a possible association between CA-125 and histological grade, stage, lymph node metastases, MI, and cervical involvement in EC, the clinical utility of CA-125 as a marker for EC has yet to be established^13^.

Most studies of CA-125 and EC included patients with advanced-stage disease^8-10^. Therefore, CA-125, as an epithelial surface antigen, can be expected to be elevated in patients with advanced-stage disease and may be associated with disease recurrence. Limited studies on low-risk and early-stage EC patients have produced conflicting results, leading to a lack of clarity in the findings.

In a multicenter retrospective study, Kim et al. found a significant association between elevated CA-125 levels and poor survival rates in patients with FIGO stage 1–2 EC^16^. In a prospective study with low-grade EC patients (n=240), disease recurrence was significantly higher in patients with elevated preoperative CA-125 levels compared with those with normal levels (19.4% vs. 7.9%, p=0.028)^7^. Logistic regression analysis identified age, tumor grade, LVSI positivity, and CA-125 levels as significant factors affecting DFS^7^. However, another study failed to find a relationship between preoperative serum CA-125 levels and disease recurrence in EC^17^. This study focused on early-stage EC patients with endometrioid histology and found an association between CA-125 levels and disease recurrence in this group of patients.

Studies investigating the role of CA-125 levels in predicting EC prognosis have reported varying thresholds^16-18^. Chen et al. established a cut-off level of 40 IU/mL for predicting disease relapse in stage 1 EC^18^. In another study, the cut-off values for CA-125 were determined to be between 15.3 and 22.9 IU/L for factors such as MI, cervical invasion, lymph node metastasis, LVSI, and disease recurrence^19^. We performed receiver operating characteristic (ROC) analysis to determine the CA-125 threshold for predicting disease recurrence and found that levels above 20.05 IU/mL had a specificity of 76.9% and a sensitivity of 68.4% for detecting recurrence risk (AUC: 0.714, 95%CI 0.59–0.83, p=0.002). However, this cut-off value was non-significant for other factors such as tumor grade, tumor size, MI, cervical invasion, and LVSI. Thus, we used the cut-off as 35 IU/mL in our study.

In EC patients, LVSI positivity is known to be an independent risk factor for disease recurrence^5,20^. However, a study by Bendifallah et al. failed to show a statistically significant relationship between LVSI and disease recurrence in low-risk EC patients (n=213), where only 10.4% were positive for LVSI^21^. The authors attributed the lack of significance to the low incidence of LVSI in the low-risk patient subset^21^. In this study, LVSI positivity was present in 16% of subjects, and we found a significant association between LVSI positivity and disease recurrence in early-stage EC.

In addition to LVSI, tumor size and MI depth are also considered risk factors for poor prognosis in EC^13,22^. However, the optimal tumor size for determining the risk of recurrence in low-risk EC is still unclear^23-25^. In a retrospective survival analysis of 720 patients, Ureyen et al. did not find a statistically significant difference in disease-free survival rates between patients with tumor size ≥35 vs. <35 mm (96.6 vs. 100%; p=0.102)^23^. In contrast, a multicenter study of 302 low-risk EC patients reported a significant difference in recurrence rates between patients with tumor size ≥35 vs. <35 mm (1 vs. 8%, p=0.006)^24^. Yet another study that used a cutoff of 2 cm for tumor size found no difference in recurrence-free survival rates of the stage 1 EC patients (HR 0.702, 95%CI 0.302–1.629, p=0.41)^25^. In our study, we did not find any association between tumor size >2 cm and disease recurrence or CA-125 levels. In the field of EC, MI is considered a crucial factor in determining a patient’s risk profile^13^. In a prospective study, Kim et al. found a significant association between high levels of CA-125 and a higher rate of MI>50%^7^. Our results revealed that patients with elevated CA-125 tended to have higher rates of MI>50%, but the difference was not statistically significant.

Various molecular changes, including genetic mutations, can play a significant role in influencing the prognosis of EC. Specifically, the current staging system for EC places a specific emphasis on certain genetic mutations, highlighting their importance among the myriad molecular alterations that impact the prognosis of EC^26^. Ongoing research in this field is shedding light on potential risk factors. For instance, Giordana et al. have suggested that polyps characterized by the hyperexpression of MKI67 and BCL2 may pose a potential risk for EC^27^. Additionally, in a study involving women with polycystic ovary syndrome (PCOS), it has been discussed that the increased risk of endometrial hyperplasia and malignancy in PCOS may be linked to decreased CASP3 (Caspase-3) activity in these patients^28^. Further exploration of these molecular signatures holds the potential to deepen our understanding of the underlying mechanisms and facilitate the development of targeted preventive strategies in the context of EC.

This study has limitations including retrospective design and single-center data, as well as the absence of follow-up CA-125 levels. Additionally, not performing LND in all patients could result in an underestimation of the stage of EC, which is another limitation. Despite these limitations, the relatively large number of patients, only early-stage diseases being studied, exclusion of adnexal masses as they may cause elevation of CA-125, and similar demographic data between groups are the strengths of this study.

## CONCLUSION

Preoperative elevation of CA-125 levels may predict a poor prognosis and decreased DFS in patients with early-stage EC. Therefore, preoperative evaluation of CA-125 can be used as an additional tool, alongside MI or tumor size, to determine the risk in these patients. However, further prospective studies are needed to validate these findings.
